# Cardiac organoids do not warrant additional moral scrutiny

**DOI:** 10.1186/s12910-024-01064-6

**Published:** 2024-05-21

**Authors:** Jannieke N Simons, Rieke van der Graaf, Johannes JM van Delden

**Affiliations:** Department of Bioethics and Health Humanities, Julius Center for Health Sciences and Primary Care, University Medical Center Utrecht, Utrecht, The Netherlands

**Keywords:** Heartbeat, Organoids, Ethics, Moral intuitions, Moral status, Organ donation, Abortion

## Abstract

Certain organoid subtypes are particularly sensitive. We explore whether moral intuitions about the heartbeat warrant unique moral consideration for newly advanced contracting cardiac organoids. Despite the heartbeat’s moral significance in organ procurement and abortion discussions, we argue that this significance should not translate into moral implications for cardiac organoids.

## Introduction

Recently, the first variations of contracting heart organoids with distinct chamber-like structures were successfully generated [1–4]. Organoids are three-dimensional in vitro cell-based models engineered out of stem cells to resemble the structure and function of small-scale organs, such as the kidney, liver, gut, and the brain [5]. Organoids are considered to be of great value to science. Heart organoids, for example, provide an unprecedented look into the first stages of heart development and the development of congenital heart defects [6]. In ethical debate, certain subtypes of organoids seem to be particularly thought-provoking, mainly brain and embryo organoids [7–9]. These cerebral and embryonic organoids are often considered to be morally distinct from other organoid subtypes, such as kidney, liver, and gut organoids.

Organoids are popularly explained and conceptualized as ‘mini-organs’, even though this practice has been criticized for tacitly overstating similarities between organoids and the organs they model [10, 11]. Nevertheless, cardiac organoids are said to “beat like the real thing” [12] and are regarded as “mini-hearts in a dish” [13]. We suspect that moral intuitions related to the heartbeat as a hallmark for life may arise with respect to cardiac organoids. Such intuitions have precedents for generating ethical, legal, and social implications, for example in the context of abortion. In Hungary, for example, it is now mandatory for pregnant persons to listen to the fetal heartbeat before terminating the pregnancy [14]. Moreover, several states in the USA have enacted ‘heartbeat bills’ that prohibit (most) abortions from the moment a fetal heartbeat can be detected (e.g., [15–17]), and the slogan “abortion stops a beating heart” is a popular rallying cry for anti-abortion activists. Though we lack empirical evidence to support our assumption that the moral significance of the heartbeat does indeed arise in cardiac organoids as it does in the context of abortion, a kindred sentiment was expressed by a participant in an empirical study on public attitudes towards research with human embryo-like structures: “the moment the heart develops, I say ‘until here and no further’” [18]. Moreover, empirical studies have shown the relationship to organoids to be ambiguous [19], with some perceiving organoids as a small parts of themselves while others consider organoids to be “a bunch of cells” [20]. Also, questions have previously arisen about the acceptability of “creating life in a dish” in relation to cerebral and embryonic organoids [21]. The interpretation of the heartbeat as hallmark for life would make the same question particularly pressing for cardiac organoids.

In this paper, we explore whether moral intuitions about the heartbeat may imply that cardiac organoids should be seen as a morally distinct subtype.

The initiation and advancement of organoid technology has raised challenging ethical issues both related to organoid technology in general and to specific subtypes [8, 9]. The first category includes concerns related to derivation of organoids, organoid usage, ownership of organoids, and storage of organoids in biobanks. For example, in organoid biobanking, where complete de-identification is considered to be undesirable because of decreased scientific and clinical value, securing informed consent is crucial but challenging: though it is common for biobank samples’ future study purposes to be undetermined during initial consent procedures, concerns arise about the inadequacy of traditional approaches for obtaining valid informed consent for organoid biobanking due to the potential sensitive uses of organoids and the ‘immortal’ cell lines involved in generating them [22].

In the second category, ethical debates mainly focus on cerebral and embryonic organoids due to their potentially elevated moral status [7–9], though the current state of organoid technology has led the International Society for Stem Cell Research (ISSCR) at present to exempt *all* organoid research from a specialized (ethical) oversight process [23]. For both organoid subtypes, challenges remain such as determining consciousness in cerebral organoids or establishing the threshold for embryonic organoids’ similarity to human embryos. Nevertheless, the central concern regarding their moral status revolves around their potential, facilitated by bioengineering, to acquire a biological feature that is deemed morally significant, distinguishing them from other organoids. Liver, kidney, or gut organoids, for example, lack such morally significant biological features.

## Normative role for the heartbeat: organ procurement and abortion

The presence of a heartbeat has had discernible normative consequences in debates on the permissibility of organ procurement and abortion.

In the Western context, the ethical hallmark of permissible vital organ procurement is the Dead Donor Rule (DDR) [24]. Essentially, the ethical consensus is that procurement of vital organs is wrong if it causes the death of the donor. To guarantee that the procurement of the organs does not cause the death of the donor, the donor must be dead at the time of the organ procurement [25]. As a determinant for the distinction between ‘alive’ and ‘dead’ the heartbeat becomes a morally significant biological feature. The intricate relationship between the DDR, determinations of death, the heartbeat, and ethically permissible organ procurement is reinvigorated and exemplified by recent debates around the ethical permissibility of thoracoabdominal normothermic regional perfusion (TA-NPR), which is a technique that allows for procurement of the heart [26]. In TA-NPR, after a patient has been declared dead based on cardiologic criteria (i.e., irreversible cessation of cardiac functioning), cardiac functioning is resuscitated. Since donor hearts for organ transplantation are particularly scarce, TA-NPR has understandably piqued the interests of medical experts. However, there are concerns that TA-NPR may violate the DDR. It has been argued that restoration of cardiac function nullifies the previously issued declaration of death [26]. The void declaration of death would mean that the patient was *not* dead at the time of procurement of the heart, and removing the heart from the patient will be what caused the death of the patient. Moreover, TA-NPR characteristically prevents perfusion to the brain, which, considering the potentially void declaration of death prior to TA-NPR, can be interpreted as causing brain death of patients [26]. Therefore, based on either cardiac death by removal of the heart or brain death by preventing perfusion of the brain, TA-NPR could be interpreted as a violation of the DDR, and, thus, morally unacceptable.

In the context of abortion, the heartbeat’s relationship with death, has been inverted to establish a relationship between the heartbeat and life: since the cessation of the heartbeat indicates death, its onset must indicate life [27]. Recently introduced anti-abortion legislation in the USA prohibits most abortions once a fetal heartbeat is detectable. Though the specifics of these ‘heartbeat bills’ differ among states that have enacted such laws, they share a common rationale: a detectable heartbeat signals that the fetus is alive and should therefore be granted the same level of moral (and legal) protection that other living members of our species enjoy [28, 29]. This new reliance on the fetal heartbeat is a divergence from the classic anti-abortionist stance, which posited that the fetus deserves moral protection from the moment of conception [30]. This is not to say that ‘moment of conception’-arguments are forsaken. On the contrary, just recently the Supreme Court of Alabama ruled that frozen embryos should be considered children, reiterating that Alabama law recognizes human life to begin at conception [31]. According to the new anti-abortion rationale, the fetus undergoes a moral transformation and becomes worthy of protection once the fetus exhibits a heartbeat [27]. The heartbeat is proclaimed as a biological feature that elevates the moral status of the entity that possesses it.

### Problematic projection onto cardiac organoids

The heartbeat’s moral significance in the context of organ procurement and abortion should not automatically translate into moral implications for cardiac organoids.

First, although the distinction between life and death is obviously morally significant, the heartbeat as determinant for this distinction is problematic. An indication that the heartbeat is an inadequate determinant to distinguish life from death is that a person can be alive *without* a heartbeat such as patients with artificial mechanical hearts. Moreover, many have argued that a person can be dead *with* a heartbeat such as patients who are declared brain dead, at least according to the common legal definition of death based on irreversible cessation of brain function [32]. The problematic nature of the ceased heartbeat as determinant for death is rather unsurprising when one considers the complexity of determining death [33]. As expressed elsewhere: “Death is a biological reality, but the means to declare death and time of death are social constructs.” [34] Medical advances such as respiratory machines, artificial hearts, and new organ procurement techniques have made it possible to save lives, but have also further complicated declaring death.

Despite the problematic nature of the heartbeat as determinant for death, anti-abortionists have inverted the heartbeat’s relationship to death in an effort to establish a relationship between the heartbeat and life. However, by borrowing the heartbeat’s relationship to death, the suggested relationship between the heartbeat and life has also inherited the problems associated with the heartbeat as determinant for death. If we accept that a person can be alive without a heartbeat - e.g., provision of an artificial mechanical heart - then the presence of a heartbeat cannot be necessary to determine life. If we accept that a person can be dead with a heartbeat - e.g., brain dead - then the presence of a heartbeat cannot be sufficient to determine life. Thus, interpretation of the heartbeat as morally significant due to its role in distinguishing life from death is problematic because the heartbeat is not convincingly apt to make this relevant distinction.

Second, the heartbeat does not fulfil the same function in the context of cardiac organoids. Critique on the heartbeat as unsatisfactory determinant of the distinction between life and death is not a suggestion to disregard the validity of the socially accepted cardiac standard of death. After all, in the absence of medical intervention, the cessation of one’s heartbeat does usually signal impending death. When one’s heart stops beating, the various systems within one’s body, as part of the larger organism, begin to falter and cease functioning: a person might not be dead just yet, but is certainly dying. This same principle of integrated functioning applies to declarations of death based on the cessation of respiratory or brain function [32, 35]. In lieu of medical intervention, the ceased function of any of these three organs, means the loss of function of the organism as an integrated whole – and thus, the loss of life in a biological sense [35]. In essence, emphasis on the importance of the heartbeat in determinations of death recognizes the heartbeat’s role in the overall functioning and survival of the organism, and we acknowledge the vital importance of this integral cardiac function.

However, a cardiac organoid is not an integrated part of a bigger whole but rather an isolated entity. Therefore, the heartbeat in cardiac organoids should not be regarded as an indication of life, interpreted as the integrated functioning of the organism. Instead, these “mini-hearts in a dish” are closer analogous to a “heart-in-a-box”, which is a new device that mimics the human body to keep the heart viable for transplantation [26]. In this “box”, the extracted heart will beat again. Nonetheless, beating hearts in isolation should not inherent associations and moral intuitions about life that are based on the heartbeat as representative of a biologically vital function in an integrated whole, an organism.

Even if we were to consider the cardiac organoid as an entity itself to comprise an integrated whole, the significance of the heartbeat in a human being still does not translate to the heartbeat in a cardiac organoid. Cardiac organoids consist of different cell types, including cardiomyocytes (i.e., cardiac muscle cells), which give cardiac organoids their contractility. If the cardiomyocytes deteriorate, the cardiac organoid will stop contracting (e.g., [36]). Now, the loss of the ability to contract is the *result*, not the cause, of the compromised function of the integrated whole (i.e., the cardiac organoid), unlike, as discussed above, the cessation of the heartbeat, which can be the *cause* of the resulting loss of functioning as an integrated whole (i.e., the organism). Moreover, considering a system composed of multiple organoids, e.g., heart, liver, and lung organoids [37], it could be argued that the cardiac organoid is an integrated part of bigger whole. However, if and only if the contraction fulfils a similarly vital function for maintaining integrated functioning of the whole, could cardiac organoids inherent associations and moral intuitions about life that are based on the heartbeat.

Third, it can be argued that cardiac organoids do not even have a heartbeat, which precludes the translation of the heartbeat’s moral significance from the context of organ procurement and abortion into moral implications for cardiac organoids both in isolation as well as in an integrated multi-organoid system. Typically, the heartbeat is understood as the pulsation of a heart. Essentially, no heart means no heartbeat. Cardiac organoids might beat *like* the real thing, but they are not the ‘real thing’ itself. Therefore, it could be claimed that any moral argument based on the heartbeat would be void for cardiac organoids as they are not hearts but instead merely incomplete models of a heart.

Similarly, opposers of the ‘heartbeat bills’ have raised the point that what is detected in the embryo is not actually a heartbeat [16]. The electrical pulsations detected in an embryo at the early stages of gestation are not produced by a heart: the embryo has not developed a heart yet, only the beginning structures from which the heart later arises. However, proponents of the ‘heartbeat bills’ have argued that embryos might indeed not have a heart yet, but they will develop one. Although, cardiac organoids are the structural equivalent of the cardiac structures in the embryo at an early state, this defensive potentiality argument does not translate from embryos to cardiac organoids, for cardiac organoids are (at present) not intended nor capable to fully develop into human hearts.

## Conclusion

While the heartbeat is deemed morally significant in discussions related to organ procurement and abortion, we have argued that similar arguments do not work in the context of cardiac organoids and do not translate into moral implications for cardiac organoids. Unless other grounds for moral consideration are articulated, cardiac organoids do not warrant additional moral scrutiny and should not be classified as a morally distinct subtype of organoids, despite moral intuitions about the heartbeat. Ultimately, the heart of ethical evaluation lies in moral considerations that go beyond intuitions. Moral intuitions should be the start of ethical exploration and moral evaluation, not the end of it.

## Data Availability

Not applicable.

## Abbreviations

ISSCR: International Society for Stem Cell Research
DDR: dead donor rule
TA-NPR: thoracoabdominal normothermic regional perfusion
